# A whole‐brain modeling approach to identify individual and group variations in functional connectivity

**DOI:** 10.1002/brb3.1942

**Published:** 2020-11-18

**Authors:** Yi Zhao, Brian S. Caffo, Bingkai Wang, Chiang‐Shan R. Li, Xi Luo

**Affiliations:** ^1^ Department of Biostatistics Indiana University School of Medicine Indianapolis IN USA; ^2^ Department of Biostatistics Johns Hopkins Bloomberg School of Public Health The University of Texas Health Science Center at Houston Houston TX USA; ^3^ Department of Psychiatry Yale School of Medicine The University of Texas Health Science Center at Houston Houston TX USA; ^4^ Department of Neuroscience Yale School of Medicine The University of Texas Health Science Center at Houston Houston TX USA; ^5^ Department of Biostatistics and Data Science The University of Texas Health Science Center at Houston Houston TX USA

## Abstract

Resting‐state functional connectivity is an important and widely used measure of individual and group differences. Yet, extant statistical methods are limited to linking covariates with variations in functional connectivity across subjects, especially at the voxel‐wise level of the whole brain. This paper introduces a modeling approach that regresses whole‐brain functional connectivity on covariates. Our approach is a *meso*scale approach that enables identification of brain subnetworks. These subnetworks are composite of spatially independent components discovered by a dimension reduction approach (such as whole‐brain group ICA) and covariate‐related projections determined by the covariate‐assisted principal regression, a recently introduced covariance matrix regression method. We demonstrate the efficacy of this approach using a resting‐state fMRI dataset of a medium‐sized cohort of subjects obtained from the Human Connectome Project. The results suggest that the approach may improve statistical power in detecting interaction effects of gender and alcohol on whole‐brain functional connectivity, and in identifying the brain areas contributing significantly to the covariate‐related differences in functional connectivity.

## Graphical abstract

This paper introduces a modeling approach that regresses whole‐brain functional connectivity on covariates. Our approach enables the identification of brain subnetworks, which are composite of spatially independent components discovered by a dimension reduction approach (such as whole‐brain group ICA) and covariate‐related projections determined by the covariate‐assisted principal regression, a recently introduced covariance matrix regression method. Applying to the Human Connectome Project data, the results show that the approach enjoys improved statistical power in detecting interaction effects of sex and alcohol on whole‐brain functional connectivity, and in identifying the brain areas contributing significantly to the covariate‐related differences in functional connectivity.

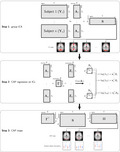

## 1 INTRODUCTION

In experiments of resting‐state functional magnetic resonance imaging (fMRI), the study of connectivity to characterize cerebral functional segregation and functional integration has received considerable attention. The understanding of brain functional organization may provide critical insights to cognitive function, as well as mental diseases. Functional connectivity, defined as the correlation or covariance between fMRI time courses, reveals the level of synchrony in the fluctuations of blood oxygenation‐level‐dependent (BOLD) signals between brain regions (Friston, 1994). Brain regions with high functional connectivity are generally grouped as functionally related and defined as a functional module/subnetwork. For example, the default mode network (DMN) is a functional subnetwork that shows greater activity during resting states than during many task challenges, and has been consistently identified through resting‐state functional connectivity analysis (Greicius et al., 2003). Existing literature has shown that brain functional connectivity varies with respect to individual characteristics, such as sex and age (Lopez‐Larson et al., 2011; Scheinost et al., 2015; Zhang et al., 2016), and in patients with autism spectrum disorders (Assaf et al., 2010), Alzheimer's disease (Wang et al., 2007), schizophrenia (Lynall et al., 2010), and other psychiatric disorders, as compared to healthy controls.

To describe group‐level differences in brain functional connectivity, investigators typically perform statistical analysis on each individual connection. One critical drawback of this element‐wise approach is the issue of multiplicity. That is, with p brain voxels/regions, statistical inference needs to account for at least $p\left(p‐1\right)/2$ hypothesis tests, one for each matrix element. To circumvent this, Zhao et al. (2019) proposed a whole‐matrix regression approach called covariate‐assisted principal (CAP) regression. It aims to identify a common linear projection of p time courses across subjects so that variations in functional connectivity defined by the projection can be explained by the covariates of interest. It is considered as a *meso*scale approach in the sense that with an appropriate thresholding, the projection defines a brain subnetwork. However, this approach suffers from the so‐called “curse of dimensionality,” in that the dimension of the data, p, cannot be greater than the number of fMRI volumes. Therefore, it cannot be applied to voxel‐level fMRI data. Other examples include a network‐based statistic proposed in Zalesky et al. (2010) and a connectome‐based pipeline introduced in Shen et al. (2017). However, both approaches are for ROI‐based networks that are constructed from (thresholded) connectivity matrices, and they can be hard to scale to large networks at the voxel level. They also have different aims from ours. Zalesky et al. (2010) employed a hypothesis‐driven approach to reduce the dimensionality of brain networks while we will develop a data‐driven approach building on Zhao et al. (2019). Shen et al. (2017) studied the inverse prediction problem than ours, where they used brain connectivities to predict demographic/clinical factors.

In this study, we propose an approach, which can be directly applied to whole‐brain voxel‐level data, revealing individual and group variations in functional connectivity. The proposed approach is also a *meso*scale approach as it identifies brain subnetworks shared across subjects. These subnetworks are composite of spatially independent components discovered by group independent component analysis (ICA Calhoun et al., 2001) and covariate‐related projections determined by the CAP regression. In fMRI studies, ICA is a widely used technique to cluster brain voxels into subnetworks (Beckmann, 2012). Applied to resting‐state fMRI data, spatial ICA identifies spatially independent and temporally coherent components. Based on the biological assumptions regarding spatial contiguity of brain networks across individuals, group ICA was introduced for population‐level studies (Calhoun et al., 2001). Other methods that identify common components in large scale data—group principal component analysis (PCA, Smith et al., 2014) and template ICA (Mejia et al., 2019), for instance—can also be applied to this end.

This paper is organized as follows. In Section 2, we introduce our proposed approach. Section 3 presents an application in resting‐state fMRI data obtained from the Human Connectome Project (HCP). Section 4 summarizes results with a discussion.

## 2 METHOD

Let $Y_{i}={\left(y_{i1},\ldots ,y_{iT_{i}}\right)}^{T}\in R^{T_{i}\times V}$ denote the $T_{i}$ BOLD scans of V voxels acquired from subject i (i=1,…,n, n is the number of subjects) in the resting‐state fMRI study, where $y_{\mathrm{it}}={\left(y_{it1},\ldots ,y_{\mathrm{itV}}\right)}^{T}\in R^{V}$ is a random variable with mean zero and covariance matrix $\Phi_{i}={\left(\phi_{\mathrm{ijk}}\right)}_{j,k}\in R^{V\times V}$. We assume that $Y_{i}$ satisfies the following decomposition:

(1) $$Y_{i}=A_{i}S,$$

where $A_{i}={\left(a_{i1},\ldots ,a_{iT_{i}}\right)}^{T}\in R^{T_{i}\times K}$ is the scalar mixing matrix and $S\in R^{K\times V}$ is the spatial component maps shared across subjects. Model (1) approximates the real fMRI data with a low‐rank matrix (Calhoun et al., 2001; Smith et al., 2014). One classic way of analyzing above group ICA data is to firstly obtain the Pearson correlation between the ICs, that is the correlation between the columns of $A_{i}$. Then, the correlations are Fisher z‐transformed and fit in a linear regression model. This is conducted on $K\left(K‐1\right)/2$ pairs of correlations; thus, the p‐values are corrected for multiplicity usually following procedures such as the Benjamini–Hochberg procedure to control for the false discovery rate (Benjamini & Hochberg, 1995). In this study, we propose an approach, where only R (<K) regression models will be fitted, which significantly reduces the number of the coefficient parameters to be estimated. In addition, the proposed approach enables a new decomposition of the brain, and each brain map is related to a set of covariates. In comparison, the proposed method decomposes the signals into R components that are associated with the covariates, which significantly alleviates the multiplicity issue and thus can improve statistical power.

For $A_{i}$ in (1), it is assumed that $a_{\mathrm{it}}\in R^{K}$ is normally distributed with mean zero and covariance matrix $\Sigma_{i}$, that is

(2) $$\text{vec}\left(A_{i}\right):N\left(0,\Sigma_{i}⊗I_{T_{i}}\right),\Sigma_{i}=\Gamma_{i}\Lambda_{i}\Gamma_{i}^{T}=\sum_{k=1}^{K}\lambda_{\mathrm{ik}}\gamma_{\mathrm{ik}}\gamma_{\mathrm{ik}}^{T},$$

where $\text{vec}\left(\cdot \right)$ denotes the vectorization of a matrix; ⊗ is the Kronecker product operator; and $I_{T_{i}}$ is the $T_{i}$‐dimensional identity matrix. For i=1,…,n, it is assumed that there exist R (1≤R≤K) indices, denoted as ${\left\{c_{i}\right\}}_{c=1}^{R}$, such that, $\gamma_{ic_{i}}=\gamma_{c}$, and the corresponding eigenvalue $\lambda_{ic_{i}}$ satisfies the following log‐linear model,

(3) $$\text{log}\left(\lambda_{ic_{i}}\right)=x_{i}^{T}\beta_{c}.$$

The covariance matrix,$\Sigma_{i}$, assumes to have the eigendecomposition as presented in (2), where $\Gamma_{i}\in R^{K\times K}$ is an orthonormal matrix such that $\gamma_{\mathrm{ij}}^{T}\gamma_{\mathrm{ik}}=1$ if j=k and zero otherwise; and $\Lambda_{i}=\text{diag}\left\{\lambda_{i1},\ldots ,\lambda_{\mathrm{iK}}\right\}$ is a diagonal matrix with $\lambda_{\mathrm{ij}}$ to be the corresponding eigenvalue. Denote $\Gamma =\left(\gamma_{1},\ldots ,\gamma_{R}\right)\in R^{K\times R}$ as the R columns that are common across subjects and $U_{i}\in R^{K\times \left(K‐R\right)}$ the remaining unique columns. Reorganizing the columns in $\Gamma_{i}$,$\Gamma_{i}=\left(\Gamma ,U_{i}\right)$. For the eigenvalues of the R common components, we consider a log‐linear model (3) with the covariates of interest, where $x_{i}\in R^{q}$ is a vector of covariates collected from subject i with the first element one for the intercept, and $\beta_{c}\in R^{q}$ is the model coefficient forc=1,…,R. Let$Z_{i}=A_{i}\Gamma ={\left(z_{i1},\ldots ,z_{iT_{i}}\right)}^{T}\in R^{T_{i}\times R}$, then $z_{\mathrm{it}}\in R^{K}$ follows a normal distribution with mean zero and covariance matrix$\Lambda_{i}$. Plugging into (1),

(4) $$Y_{i}=A_{i}S\approx Z_{i}\Gamma^{T}S@Z_{i}\Omega ,$$

where the data are approximated by the R common components, and each row of $\Omega =\Gamma^{T}S\in R^{R\times V}$ represents a spatial brain map. Let $\omega_{c}={\left(\omega_{c1},\ldots ,\omega_{\mathrm{cV}}\right)}^{T}\in R^{V}$ denote the c th row of the inverse loading matrix, that is, $z_{\mathrm{itc}}=\omega_{c}^{T}y_{\mathrm{it}}$, for $t=1,\ldots ,T_{i}$. Under model (3),

$$\exp\;\left(x_{i}^{T}\beta_{c}\right)=\lambda_{ic_{i}}=\text{Var}\left(z_{\mathrm{itc}}\right)=\text{Var}\left(\omega_{c}^{T}y_{\mathrm{it}}\right)=\sum_{j=1}^{V}\omega_{\mathrm{cj}}^{2}\;\phi_{\mathrm{ijj}}+\sum_{j\neq l}\omega_{\mathrm{cj}}\omega_{cl\;\;}\phi_{\mathrm{ijl}}$$

In resting‐state fMRI studies, time courses are generally standardized to have identical standard deviation with $\phi_{\mathrm{ijj}}=\phi_{i}^{2}=\phi^{2}$, for j=1,…,V and i=1,…,n. Variations in functional connectivities are then captured by the variations in $\text{Var}\left(\omega_{k}^{T}y_{\mathrm{it}}\right)$ through a linear combination weighted by $\omega_{\mathrm{kj}}$'s. Our goal is to identify the weights (the spatial maps) Ω as well as the model coefficient $\beta_{k}$'s. In this sense, our proposed approach is a network‐level/*meso*scale analysis, where the network consists of voxels that contribute largely to the combination.

Model (1) presents one specific way of decomposing the voxel‐level fMRI data using group ICA in this paper. By modifying S as an initial decomposition, one can apply the proposed method to other types of brain parcellation. For example, in a seed‐based or a region‐of‐interest‐based (ROI‐based) analysis, each row of S is a vector of zeros and ones, where one indicates that the corresponding voxel is part of the seed/ROI. In this case, Ω clusters the seeds/ROIs into covariate‐related groups. For example, in Zhao et al. (2019), the CAP approach grouped the DMN ROIs into components related to gender difference and gender and age interactions.

### 2.1 Interpretation of the proposed model under a special case

The pairwise regression approach (Wang et al., 2007) can be considered as a special case of the proposed approach with the corresponding $\omega_{\mathrm{cj}}$ to be $\sqrt{2}$ and the rest zero. For example, considering voxles 1 and 2, the corresponding $\omega_{c}={\left(1/\sqrt{2},1/\sqrt{2},0,\ldots ,0\right)}^{T}$. Assuming ϕ=1, with $\phi_{i12}=\phi_{i21}$, we rewrite model (3) as.

$$\text{log}\left(1+\phi_{i12}\right)=x_{i}^{T}\beta_{c}.$$

The Fisher z‐transformation takes the formula

$$z=\frac{1}{2}\text{log}\left(\frac{1+r}{1‐r}\right),$$

where r is the Pearson correlation. When $\phi_{i12}\approx 0$, we have

$$\text{log}\left(1+\phi_{i12}\right)\approx \text{log}\left(\frac{1+\phi_{i12}}{1‐\phi_{i12}}\right).$$

Therefore, model (3) is approximately equivalent to the pairwise regression under this special case.

### 2.2 Algorithm

**Figure 1:**
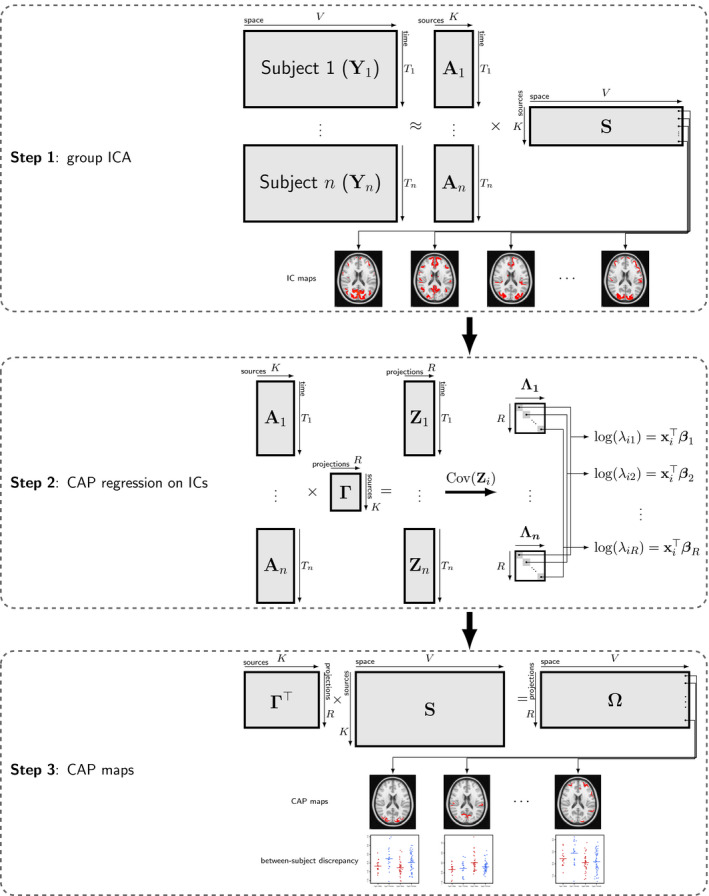
Algorithm. Step 1: group independent component analysis (ICA) on the whole brain. Step 2: the covariate‐assisted principal (CAP) regression on the IC time courses to identify projections of the ICs that are associated with the covariates of interest. Step 3: reconstruction of the CAP brain maps

To estimate the spatial mapΩ, we propose to estimate S and Γ in two steps. Figure 1 demonstrates the estimation procedure in each step. In the first step, S, which contains the spatially independent components shared across subjects, can be estimated through group independent component analysis (ICA, Calhoun et al., 2001) by temporally concatenating BOLD time courses from multiple subjects. In practice, in order to reduce the computation complexity and the amount of required memory, multiple data reduction steps, typically using principal component analysis (PCA), are performed before concatenating the time courses (Calhoun et al., 2009). After acquiring IC time courses through dual regression, Γ and$\beta_{c}$, for c=1,…,R, can be simultaneously identified using the covariate‐assisted principal (CAP) regression approach proposed in Zhao et al. (2019), where Γ contains the R common components and R is determined based on a metric that measures the level of deviation from diagonality (DfD) of the rotated matrix ${\hat{\Lambda }}_{i}={\hat{\Gamma }}^{T}{\hat{\Sigma }}_{i}\hat{\Gamma }$, where $\hat{\Gamma }$ is the estimate of Γ and ${\hat{\Sigma }}_{i}$ is an estimate of$\Sigma_{i}$, for example, the sample covariance matrix of $A_{i}$, fori=1,…,n.

(5) $$DfD\left(\hat{\Gamma }\right)=\prod_{i=1}^{n}{\left(\frac{\det\left\{\text{diag}\left({\hat{\Lambda }}_{i}\right)\right\}}{\det\left({\hat{\Lambda }}_{i}\right)}\right)}^{T_{i}/\sum_{i\;}T_{i}},$$

where $\text{diag}\;({\hat{\Lambda }}_{i})$ is a diagonal matrix with the diagonal elements the same as in ${\hat{\Lambda }}_{i}$; and $\text{det}\;\left({\hat{\Lambda }}_{i}\right)$ is the determinant of ${\hat{\Lambda }}_{i}$. If $\hat{\Gamma }$ is a common diagonalization of ${\hat{\Sigma }}_{i}$'s; that is, ${\hat{\Lambda }}_{i}$'s are diagonal matrices, the above metric is one. As suggested in Zhao et al. (2019), one can plot the metric over the number of components and choose R before the metric grows far away from one or before a sudden jump. The details of the CAP procedure are described in Zhao et al. (2019), and the implementation can be accomplished using the cap package in the open source software R. Thus, we do not repeat the algorithm in detail in this manuscript. The last step is the reconstruction of the brain maps $\hat{\Omega }={\hat{\Gamma }}^{T}\;\;\hat{S}$, called CAP brain maps. Each CAP brain map, after thresholding, should be interpreted as major brain areas contributing to specific functional connectivity variations explained by the covariates, especially those areas with statistically significant regression coefficients.

### 2.3 Inference

To draw inference on the model coefficients, it is considered to acquire the confidence intervals from bootstrap samples. In each iteration, $A_{i}$'s are resampled with replacement and fitted into Step 2 in Figure 1. Applying each column of $\hat{\Gamma }$, we estimate the corresponding β using the resampled data. This procedure is repeated for B times, and 100(1‐α)% confidence intervals are constructed, where α is the significance level, for example, α=0.05.

## 3 ANALYSIS OF RESTING‐STATE FMRI FROM THE HUMAN CONNECTOME PROJECT

We apply our proposed approach to the Human Connectome Project (HCP) resting‐state fMRI data (scan session REST1_LR). The HCP aims to characterize human brain structure, function, and connectivity, as well as their variability in healthy adults. We use the group ICA data from the HCP, as available at http://www.humanconnectomeproject.org/. The fMRI data were first minimally preprocessed following Glasser et al. (2013). The artifacts were removed by using ICA + FIX (Griffanti et al., 2014; Salimi‐Khorshidi et al., 2014). Group‐PCA results were first generated by MIGP (MELODIC's Incremental Group‐PCA) from 820 subjects, and then fed into group ICA using FSL (https://fsl.fmrib.ox.ac.uk/fsl/fslwiki/FSL) MELODIC tool (Beckmann & Smith, 2004; Hyvarinen, 1999). Spatial ICA was acquired in grayordinate space (surface vertices plus subcortical gray matter voxels; Glasser et al., 2013) at various dimensionalities.

In this study, we use the 25‐IC data of 109 subjects (aged 22–36) from the HCP S500 release. The goal is to discover brain networks, within which the functional connectivity varies due to alcohol use, and to examine whether the alcohol‐induced variation differs by gender. We apply the proposed method (i.e., ICA‐CAP) and compare with an edge‐wise regression approach. In both approaches, the regression model includes age (continuous, mean 29.0, *SD* 3.4), gender (binary, 41 females and 68 males), alcohol drinker (binary, 67 nondrinkers and 42 drinkers), and a gender × alcohol interaction (27 female nondrinkers, 40 male nondrinkers, 14 female drinkers, and 28 male drinkers) as the covariates. In the following, we will focus on the four contrasts derived from the gender × alcohol interaction; that is, (1) male versus. female among alcohol nondrinkers; (2) male versus. female among alcohol drinkers; (3) alcohol drinkers versus. nondrinkers in the female group; and (4) alcohol drinkers versus. nondrinkers in the male group.

**Figure 2:**
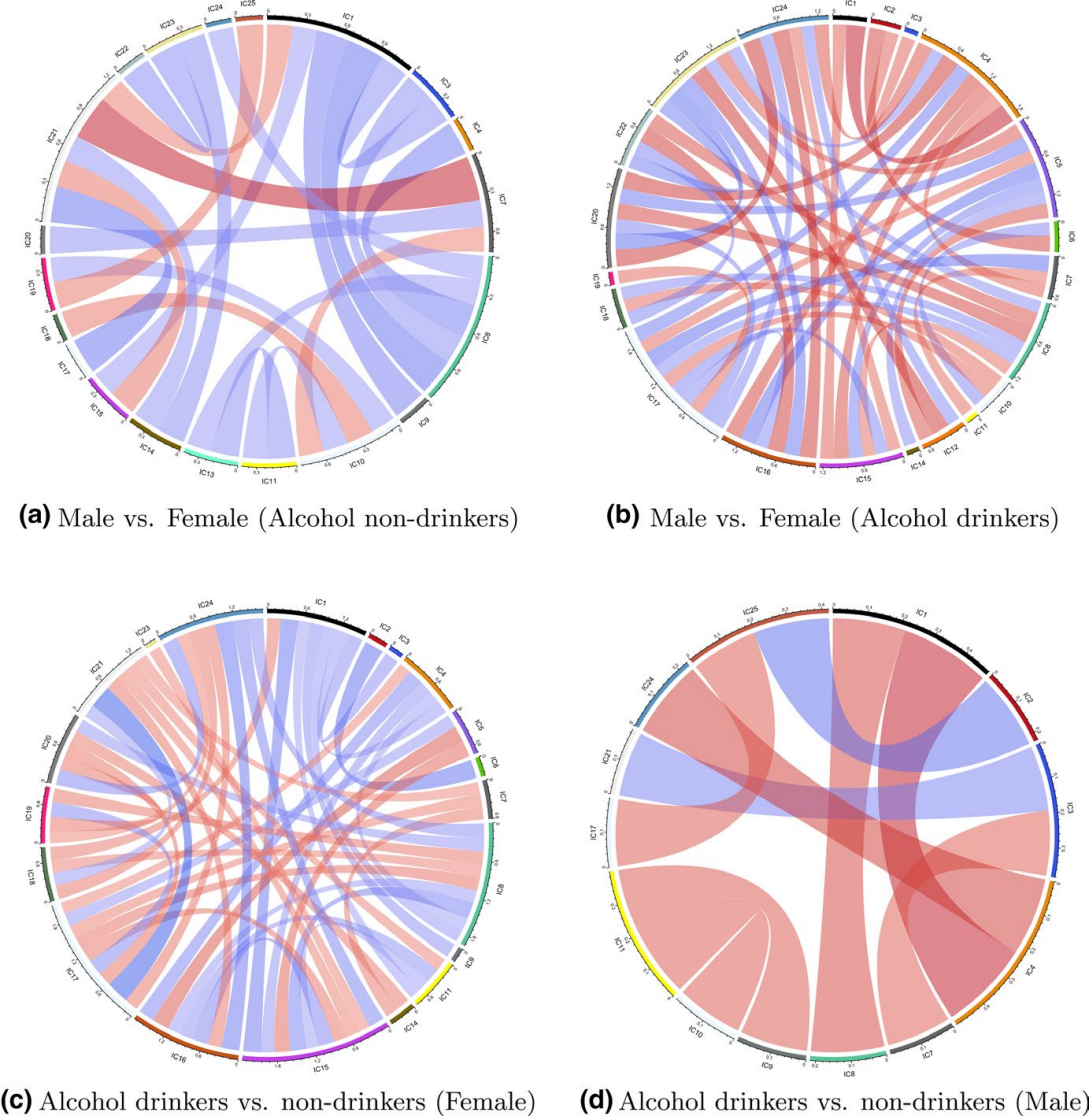
Effect size with significance of the model contrast of gender and alcohol in the edge‐wise regression. A connection indicates that the raw p‐value of the corresponding pair is <0.05. Red color indicates a positive effect, and blue indicates negative. The darkness of the color and the width of the cord suggest the magnitude of the effect. (a) Male versus Female (Alcohol nondrinkers). (b) Male versus Female (Alcohol drinkers). (c) Alcohol drinkers versus nondrinkers (Female). (d) Alcohol drinkers versus nondrinkers (Male)

In edge‐wise regression, functional connectivity between IC's is first calculated using Pearson's correlation and then Fisher z‐transformed. Linear regression is performed with the Fisher z‐transformed correlations as the outcome. We present the corresponding effect size in Figure 2 for those pairwise correlations that are significant for any contrast (at α=0.05). We observe differences in functional connectivity between IC's for all four comparisons. However, none of them survives correction for multiple testing following the false discovery rate control procedure in Benjamini and Hochberg (1995). Though the edge‐wise regression approach identifies subtle variations in functional connectivity and the interpretation is straightforward, the method suffers from the curse of dimensionality as the number of tests increases dramatically as the number of components increases.

**Figure 3:**
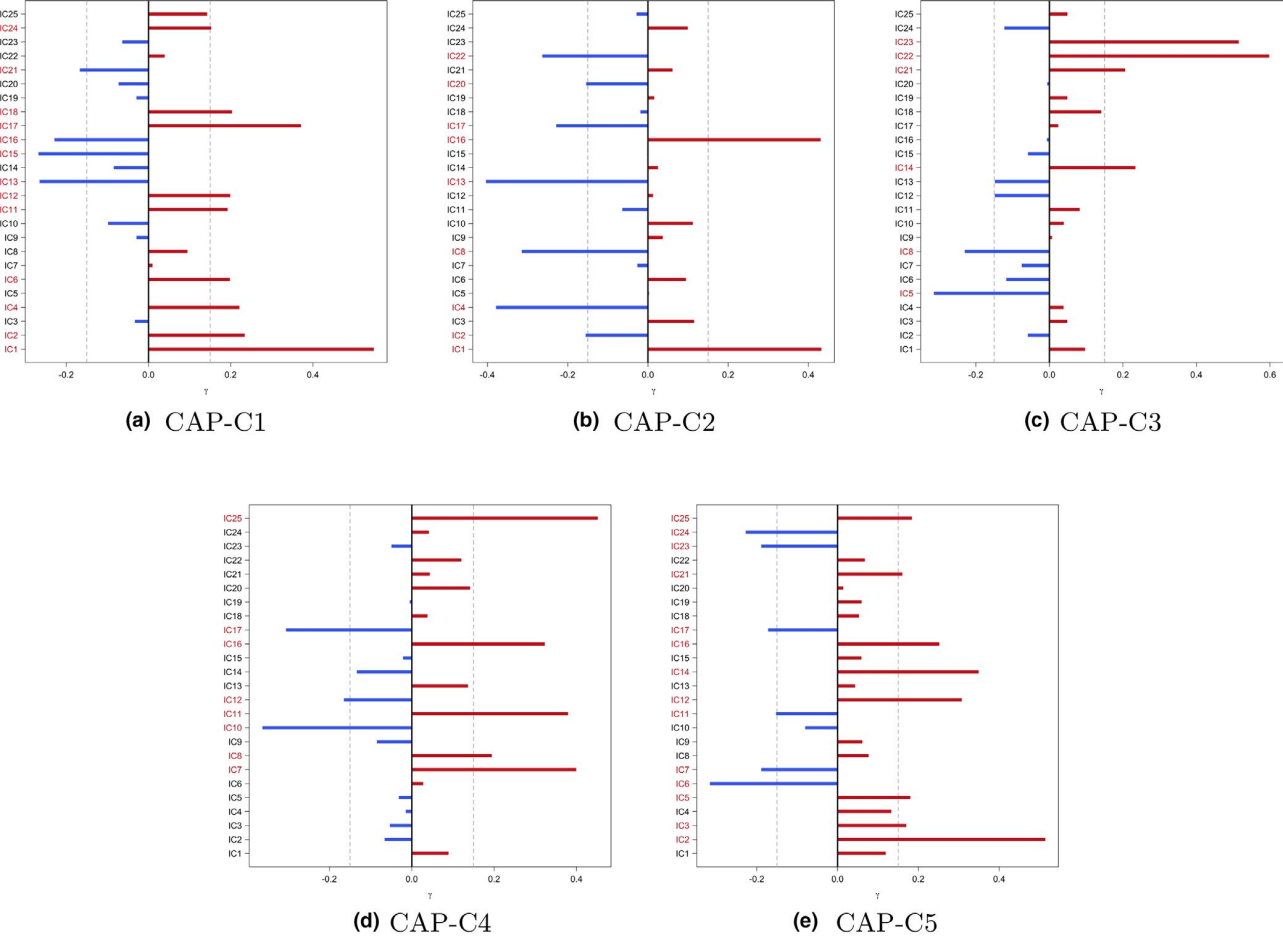
Loadings of the five identified components from the ICA‐CAP approach. ICs in red have loading magnitude >0.15 (gray dashed lines). (a) CAP‐C1. (b) CAP‐C2. (c) CAP‐C3. (d) CAP‐C4. (e) CAP‐C5

**Figure 4:**
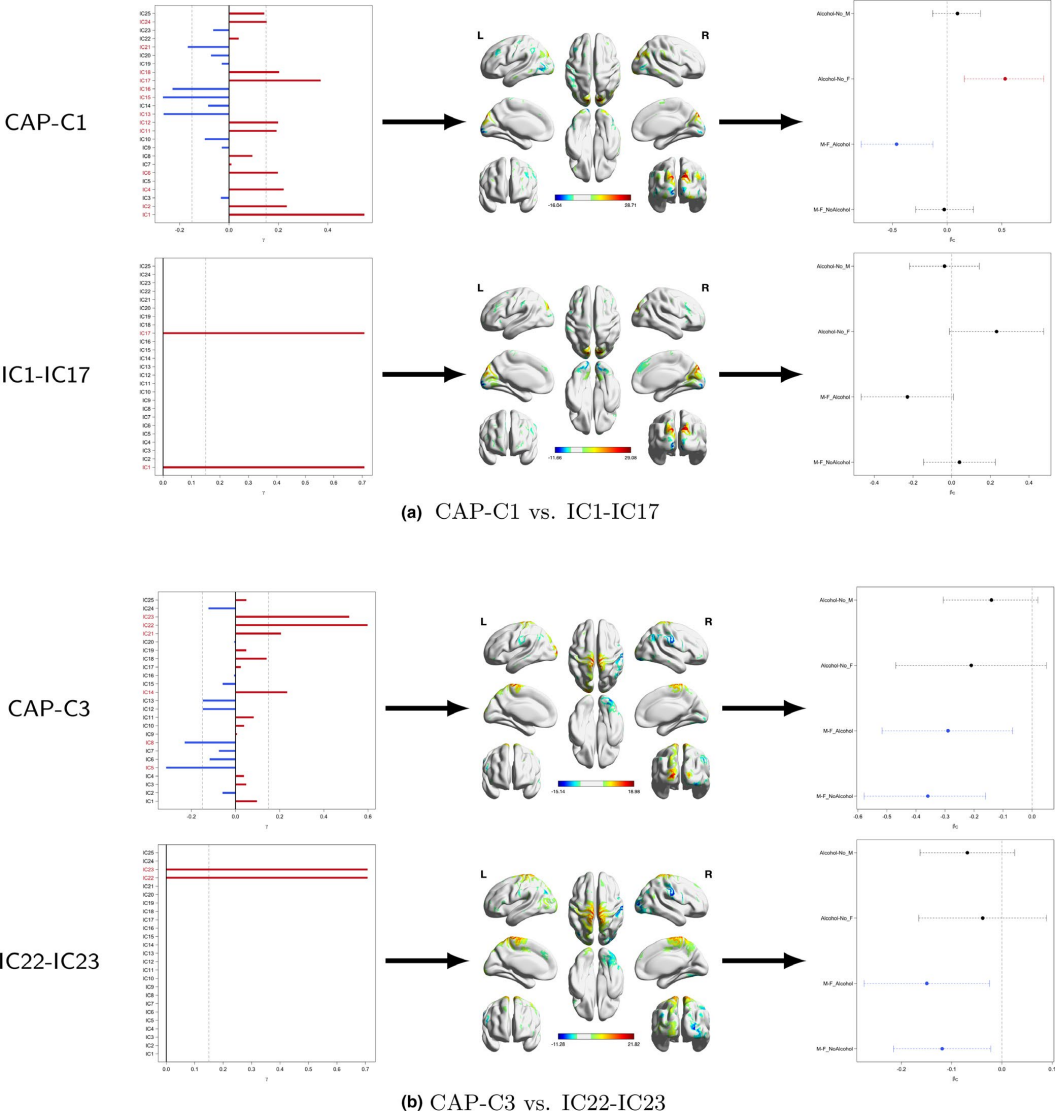
A comparison of CAP components with element‐wise regression. Figures on the left panel show the loading profile of the components/pairs, in the middle displays the corresponding brain map, and on the right presents the estimate of the four comparisons with 95% confidence intervals. For the element‐wise approach, the brain maps are superposition of the two components with equal weights. (a) CAP‐C1 versus IC1‐IC17. (b) CAP‐C3 versus IC22‐IC23

Using the deviation from diagonality to select model order, the proposed ICA‐CAP approach identifies five components. Figure 3 shows the loading profile, Table 1 displays the average percentage of variation explained by the five components, and Table 2 presents the coefficient estimates (with 95% bootstrap confidence intervals). The five identified components in total explain about 14.05\% of the data variation when averaging over all subjects. Among all five components, C4 demonstrates the largest proportion (5.73\% on average). Grouping subjects into four subgroups, we observe variations in the percentages, which are consistent with the comparisons in Table 2. For the components C1, C2, and C4, we observe significant gender difference in functional connectivity among alcohol drinkers. In addition, among females, the functional connectivity within the component network demonstrates a significant difference between alcohol drinkers and nondrinkers. For C3, both the alcohol drinkers and nondrinkers groups show significant gender difference. For C5, all four comparisons reveal significant difference in functional connectivity. Figure S1 in the Supplement presents the scatter plot of each gender × alcohol subgroup for the five components. For C1 and C3, we fit the edge‐wise regression model on the two top loading ICs and compare the results with CAP in Figure 4. Though the coefficient of alcohol in female and gender difference in the alcohol user group are marginally significant in the IC17‐IC1 pair, the direction and trend of the coefficients are consistent with the CAP component C1. For C3, the significance of the β coefficients of the top loading pair in the element‐wise regression is consistent with those in the CAP components, which verifies the ICA‐CAP findings. Here, we want to comment that the sign of the loadings in the ICA‐CAP approach is not identifiable.

**Figure 5:**
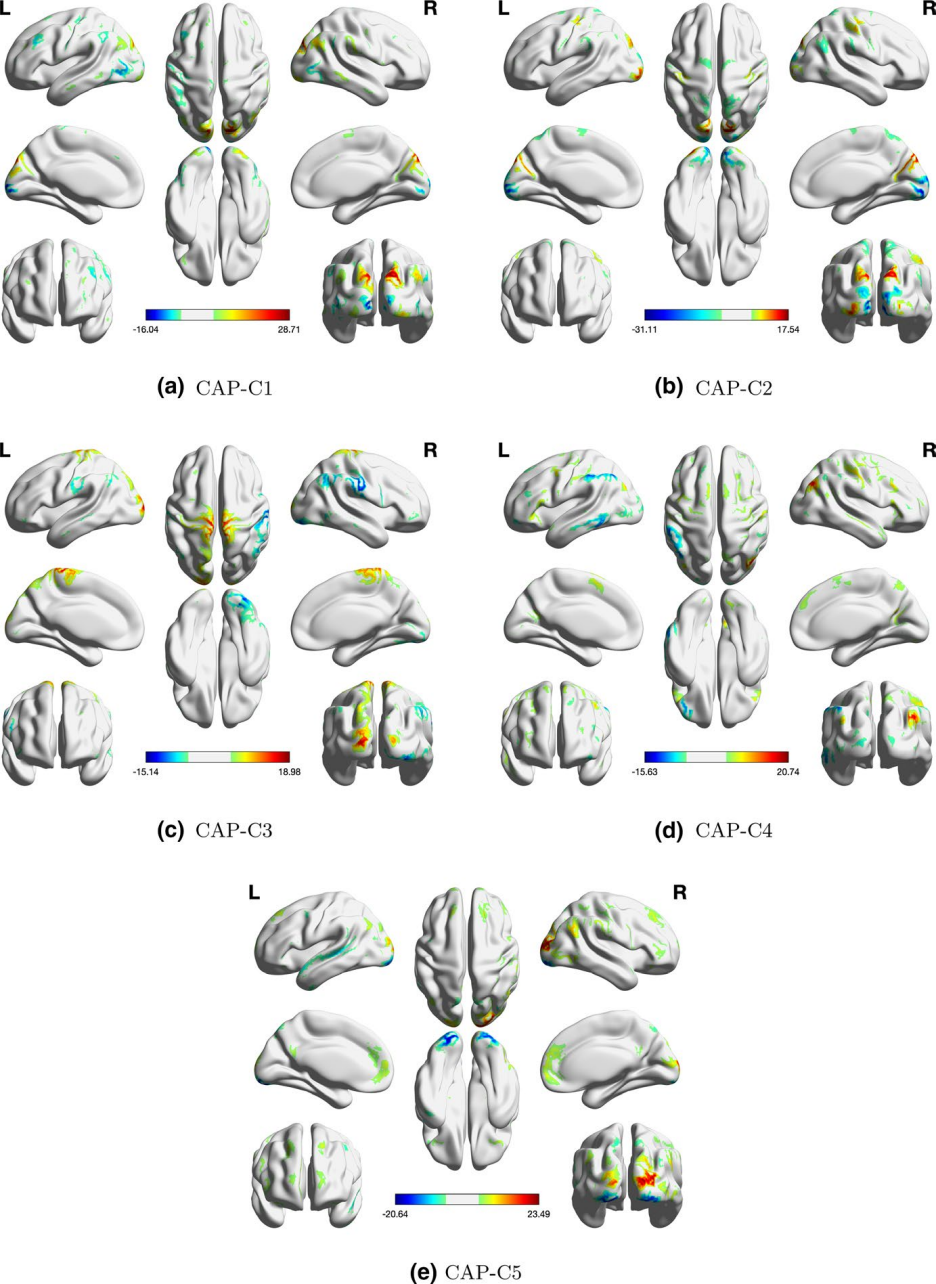
Reconstructed brain maps of the five components from the ICA‐CAP approach (cortical regions). (a) CAP‐C1. (b) CAP‐C2. (c) CAP‐C3. (d) CAP‐C4. (e) CAP‐C5

Because ICA‐based methods, including ours, measure not only the function connectivity but also interactions between brain networks (Joel et al., 2011), we thus only highlight the regions with high loadings in the reconstructed CAP brain map in Figure 5 (also see Figure S4 in the supplementary material), since these high loading components contribute majority of the variations associated with the covariates. Of the five components, we use C1 and C3 as an example to further interpret the findings. For C1, the cuneus is among the highlighted regions. The cuneus is implicated in cue‐elicited craving and altered emotion processing in alcohol misuse (Jansen et al., 2019; Jasinska et al., 2014). Compared with healthy controls, alcohol‐dependent participants showed lower degree centrality values in the cerebellum, visual cortex, and precuneus in graph theoretical connectivity analyses (Luo et al., 2017). Another connectivity study reported that the precuneus, postcentral gyrus, insula, and visual cortex were the main brain areas with reduction in network connectivity, perhaps suggesting reduced interoceptive awareness in alcohol drinkers, compared to nondrinkers (Vergara et al., 2017). Overall, the current findings do not appear to be inconsistent with these earlier reports. Nonetheless, we wish to caution that the current findings are based on the contrast between drinkers and nondrinkers whereas those of the earlier studies typically involved heavy and/or dependent drinkers. This component also suggests a gender and drinking interaction. Together, these findings add to the literature of sex differences in the neural processes underlying how alcohol and drinking variables contribute to heavier and problem alcohol use in both dependent and nondependent drinkers (Hu et al., 2018; Ide et al., 2017, 2018; Wang et al., 2019; Zhornitsky et al., 2018).

For C3, we observe gender difference in brain functional connectivity. Very few studies have examined gender differences in resting‐state functional connectivity in neurotypical populations within the age range of the current cohort. An earlier study employed ICA to identify four fronto‐parietal networks and showed sex differences in two of these networks with women exhibiting higher functional connectivity in general, an effect that appeared to be independent of the menstrual cycle (Hjelmervik et al., 2014). A lifespan study showed that the differences in connectivity between men and women of 22–25 years of age did not differ significantly in functional connectivities (Conrin et al., 2018). However, the 26–30 (p=0.003) and the 31–35 age groups (p<0.001) showed significant differences. At the most global level, areas of diverging sex difference include parts of the prefrontal cortex and the temporal lobe, amygdala, hippocampus, inferior parietal lobule, posterior cingulate, and precuneus. In a study of the elderly, males showed greater connectivity than females in the salience network, whereas females showed greater connectivity than males in the default mode network (Jamadar et al., 2018). Here, we demonstrate gender differences in somatomotor and occipital cortex, and cold color regions are the orbitofrontal cortex and temporo‐parietal junction, suggesting that the ICA‐CAP provides another analytical approach that may capture gender differences in network connectivity.

**Figure 6:**
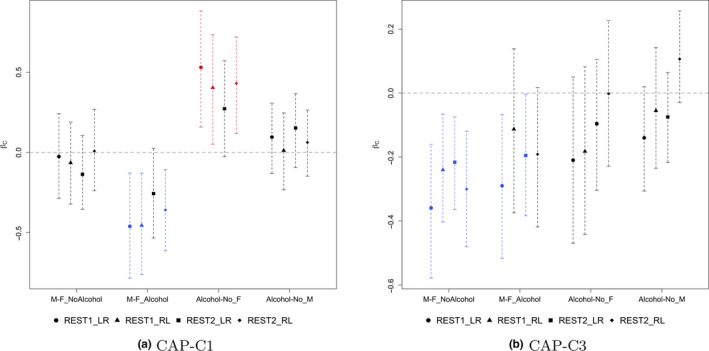
Estimated model contrast (and 95% bootstrap confidence interval) of gender and alcohol for all four fMRI scan sessions in HCP. The linear projections (CAP‐C1 and CAP‐C3) are estimated from scan session REST1_LR. Intervals in color (red for positive and blue for negative) indicate a significant effect. (a) CAP‐C1. (b) CAP‐C3

To examine the reliability of the method, we apply the identified linear projections on the rest three scan sessions of resting‐state fMRI data acquired from the same subjects and obtain the model coefficient estimates in model (3). Figure 6 presents the estimated model coefficients and 95% bootstrap confidence interval for each session, where the linear projections are estimated using the data of REST1_LR. From the figure, in CAP‐C1, the significance of the comparisons is consistent across sessions except for REST2_LR, where the effects are marginally significant. For CAP‐C3, significant sex difference among nondrinkers is observed in all four sessions. Among alcohol drinkers, sex difference is significant in sessions REST1_LR and REST2_LR and marginally significant in REST2_RL. The consistency of the findings across sessions not only demonstrates the reliability of the method, but also provides evidence of the existence of variations in functional connectivity in these brain networks. We also repeat the whole process to the rest three experimental sessions. The proposed method identifies five components in REST1_RL, six components in REST2_LR, and four components in REST2_RL. In Figure S6 of the supplementary materials, it presents the correlations between the components using a chord diagram, where a connection indicates that the correlation between the two components is over 0.5. From the figure, the correlations between the first components identified across all sessions are relatively high (>0.7), showing moderate reliability of the first component. Nonetheless, except for C5 of REST2_LR, the rest components are correlated with at least one component identified in another session. This suggests a potential limitation of the proposed approach in reproducing components across sessions.

## 4 DISCUSSION

In this study, we propose a voxel‐level approach to identify brain subnetworks that are associated with covariates of interest. The approach builds upon two technical components including a dimension reduction step and a covariance regression step. In the dimension reduction step, we consider the widely used group ICA approach to obtain spatially independent components shared by the study population. The covariance regression method identifies brain subnetworks (combinations of the components) that demonstrate population or individual variation in brain functional connectivity. The last step of the proposed approach reconstructs principal component brain maps, comprised of orthogonal groups of the ICs, in association with covariates of interest. Compared to a standard pairwise approach, which requires fitting separate models for each pair of regions/networks, the utilization of the covariance regression method illustrates superior performance by avoiding the massive number of univariate tests.

Our method comparison adds a growing literature on comparing multivariate approaches to univariate approaches for functional connectivity modeling. The effectiveness of multivariate approaches over univariate ones was also observed for multivariate covariance measures (Geerligs & Henson, 2016; Yoo et al., 2019). In this manuscript, we focused on multivariate modeling of whole correlation matrices, instead of each connectivity edge separately.

There are several methodological limitations of our current method. Given the current sample size, we did not consider functional connectivity changes over time or cognitive state, also known as dynamic connectivity (Hutchison et al., 2013). Additionally, spatial variations in functional connectivity were also found recently to be related to cognitive state (Salehi et al., 2019). Our framework takes a generalized linear model form, and this makes it amenable to inclusion of spatial and temporal covariates. Though estimating the spatial and temporal effects seems achievable, proper statistical inference would require future work to consider spatial and temporal dependence in a more complex mixed effects model framework. Secondly, our method does not model task activations and even further task‐induced connectivity changes. It marginally depends on the Gaussian distribution assumption, though Pearson's correlation is relatively robust against slight departure of Gaussianity. Note that the non‐Gaussian assumption over spatial maps in our group ICA does not apply to our Gaussian likelihood modeling of the extracted time courses. Future research is required to extend our method to accommodate various ICA approaches (Calhoun et al., 2009) with non‐Gaussian assumptions on other components. Finally, we took a multistage approach which can lead to decreased statistical efficiency. An alternative approach, though computationally more expensive, is to consider fitting CAP regression and group ICA simultaneously in a uniform model.

We apply the proposed method to the HCP resting‐state fMRI data and identify brain subnetworks within which the functional connectivity variations can be explained by gender and/or alcohol use. Our findings are in line with extant literature, lending evidence to the usefulness of the proposed method in investigating the variability in brain connectomics. The main goal of the analysis herein is to assess the effectiveness of the proposed method. Future analyses with larger cohorts are warranted to validate the findings here. With increased cohort sizes and the availability of more comprehensive covariates, the propose method may be adopted to include more complex covariates and their interactions.

We also recognize several limitations in our fMRI analysis. First, we did not evaluate variations in brain maps related to covariates. Though these maps can be useful for generating hypotheses, our current implementation does not provide statistical significance of these maps or cluster‐level p‐values. One possible direction to use bootstrapped data to evaluate the variations in recovered brain maps, though this can be computationally prohibitive not to mention a potential challenge to match brain maps across bootstrapped samples. Second, it is expected that many other covariates could impact functional connectivity networks, for example, structural imaging measures and behavioral assessments. In this first paper, we use the basic demographic variables in HCP as a demonstration of our method, and our conclusions are subject to confounding from those additional covariates not included in the model. Thirdly, we did not include additional data or external datasets to validate our findings. Here, our analysis should be treated as a confirmatory study illustrating a new method. The novel findings by our method should be further validated using ideally external data.

## 5 CONCLUSION

In this study, we propose a whole‐brain modeling approach to discover variations in brain functional connectivity. The approach can be directly applied to voxel‐level fMRI data and identifies brain subnetworks within which variations in functional connectivity are associated with population/individual covariates of interest. Applied to a resting‐state fMRI dataset obtained from the Human Connectome Project, the proposed multivariate approach is demonstrated to be effective with improved statistical power in detecting variations explained by gender and/or alcohol use.

## AUTHOR CONTRIBUTIONS

YZ and XL proposed the method. YZ conducted the analyses and drafted the paper. CRL interpreted the findings. YZ, BS, BW, CRL, and XL revised the paper critically for important intellectual content.

## ETHICAL STATEMENT

No ethical statement is required for this study.

6

**Table 1:** Average percentage of variance explained by each component from the ICA‐CAP approach. The average is calculated over all subjects as well as subjects within each subgroup of gender and alcohol interaction.

	All	Female nondrinkers	Male nondrinkers	Female drinkers	Male drinkers
C1	1.98	1.85	1.74	3.02	1.91
C2	1.64	1.67	1.58	2.21	1.42
C3	2.42	3.15	2.21	2.57	1.93
C4	5.73	5.57	6.19	4.27	5.95
C5	2.27	1.76	2.58	2.54	2.18
Total	14.05	14.01	14.32	14.61	13.41

**Table 2:** Estimated model contrast (and 95% bootstrap confidence interval) of gender and alcohol for the five identified components from the ICA‐CAP approach.

	Male vs. Female		Alcohol drinkers vs. nondrinkers	
	Alcohol nondrinkers	Alcohol drinkers	Female	Male
C1	−0.026 (−0.287, 0.241)	−0.461 (−0.785, −0.129)	0.530 (0.157, 0.882)	0.095 (−0.131, 0.307)
C2	−0.002 (−0.189, 0.202)	−0.421 (−0.624, −0.198)	0.343 (0.093, 0.585)	−0.075 (−0.247, 0.089)
C3	−0.359 (−0.579, −0.160)	−0.290 (−0.517, −0.067)	−0.209 (−0.469, 0.050)	−0.140 (−0.306, 0.020)
C4	0.014 (−0.191, 0.236)	0.289 ( 0.010, 0.559)	−0.333 (−0.602, −0.079)	−0.058 (−0.290, 0.150)
C5	0.351 (0.203, 0.534)	−0.165 (−0.336, −0.008)	0.326 (0.157, 0.496)	−0.190 (−0.368, −0.027)

### Peer Review

The peer review history for this article is available at https://publons.com/publon/10.1002/brb3.1942.

## Supporting information

Supplementary Material

## Data Availability

The group ICA data were downloaded from the Human Connectome Project, which is publicly available at http://www.humanconnectomeproject.org/. The covariate‐assisted principal regression was implemented using the R package cap available at: https://cran.r‐project.org/web/packages/cap/index.html.
