# Supplementary material for: A whole‐brain modeling approach to identify individual and group variations in functional connectivity
Source: Brain Behav. 2020 Nov 18;11(1):e01942. doi: 10.1002/brb3.1942 (PMC7821576; doi:10.1002/brb3.1942)
Supplement: Supplementary file 1 — Supplementary Material [file BRB3-11-e01942-s001.pdf]

Supplementary Materials to "A Whole-Brain Modeling Approach to Identify Individual and Group Variations in Functional Connectivity"

Yi Zhao[1,*], Brian S. Caffo[2], Bingkai Wang[2], Chiang-Shan R. Li[3,4], and Xi Luo[5]

[1]Department of Biostatistics, Indiana University School of Medicine

[2]Department of Biostatistics, Johns Hopkins Bloomberg School of Public Health

[3]Department of Psychiatry, Yale School of Medicine

[4]Department of Neuroscience, Yale School of Medicine

[5]Department of Biostatistics and Data Science,

The University of Texas Health Science Center at Houston

[*]Corresponding author: Yi Zhao, Department of Biostatistics, Indiana University School of Medicine, 410 West 10th Street, Indianapolis, IN 46202. Email: zhaoyi1026@gmail.com

A Additional Results of the Human Connectome Project Analysis

Figure A1 is the scatterplot of model outcome in the proposed ICA-CAP approach for each gender×alcohol subgroup. For example, in component C1 (Figure A1a), we observe the difference between females and males among the alcohol users; as well as the difference between female alcohol drinkers and female non-drinkers. Figure A2 is the scatterplot of the percentage of variation explained by each component. Figure A3 presents the functional connectivity matrix of two subjects with the highest and lowest model outcome in CAP-C1. One subject (Subject 82) is female and a alcohol user, and the other one (Subject 86) is also female but claims no alcohol consumption. From the figure, we observe difference in connectivity patterns , where Subject 86 has strong functional connectivities in more IC pairs.

Figure A4 provides another view of the reconstructed brain maps of the five components from the ICA-CAP approach. Regions with high positive (POS) and negative (NEG) loadings of each component are presented separately.

A.1 Comparison between methods

For C1, we fit the edge-wise regression model on the two top loading ICs, IC1 and IC15, and compare the results with CAP in Figure A5. The significance of the coefficients is consistent with the results of C1. In C1, as the sign of IC1 and IC15 are opposite, the sign of the coefficients are opposite to the edge-wise regression result.

A.2 Comparison between experimental sessions

To examine the reproducibility, the proposed approach is applied to all the four experimental sessions (REST1_LR, REST1_RL, REST2_LR and REST2_RL) in HCP. The approach identifies five components in REST1_LR, five components in REST1_RL, six components in REST2_LR, and

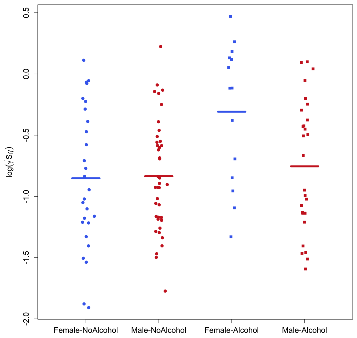

(a) CAP-C1

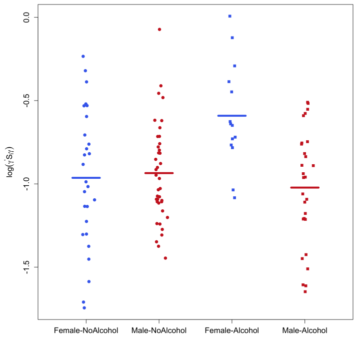

(b) CAP-C2

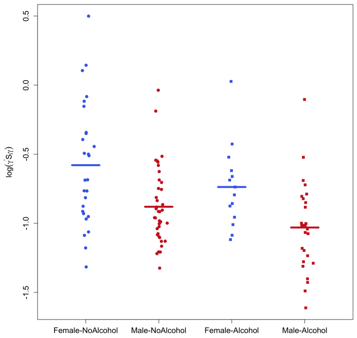

(c) CAP-C3

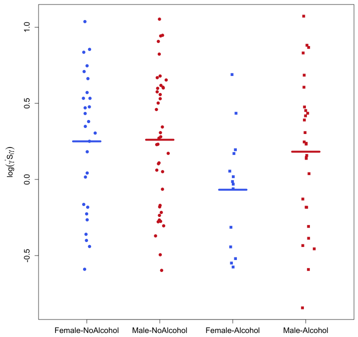

(d) CAP-C4

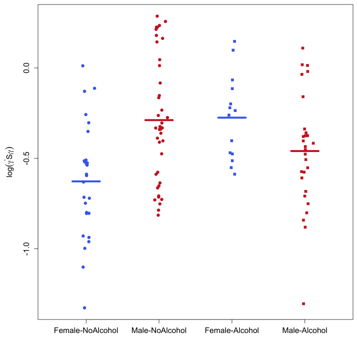

(e) CAP-C5

Figure A1: Scatterplot for the five identified components from CAP after adjusting for age. Female-NoAlcohol (blue solid circles): female alcohol non-drinkers. Male-NoAlcohol (red solid circles): male alcohol non-drinkers. Female-Alcohol (blue solid squares): female alcohol drinkers. Male-Alcohol (red solid squares): male alcohol drinkers.

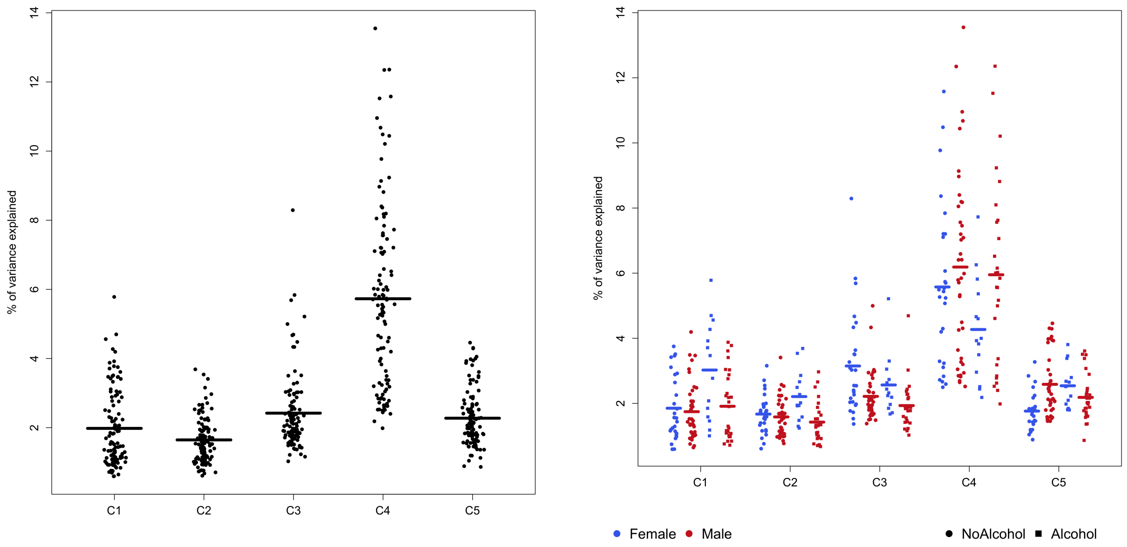

(a) All subjects

(b) By group

Figure A2: Percentage of variation explained by the five identified components from ICA-CAP. Blue solid circles: female alcohol non-drinkers; red solid circles: male alcohol non-drinkers; blue solid squares: female alcohol drinkers; blue solid squares: male alcohol drinkers.

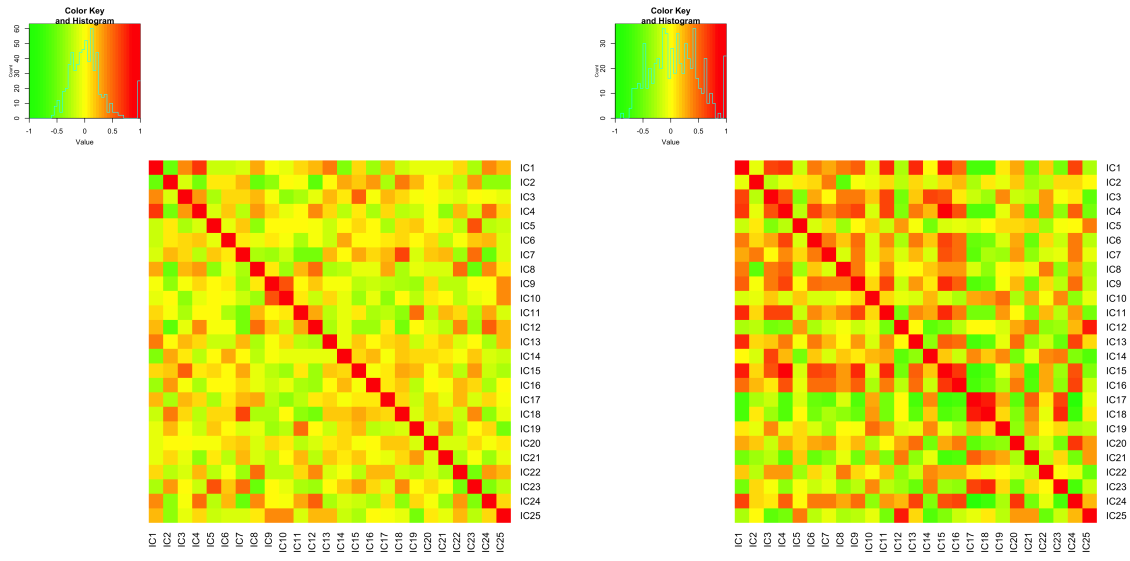

(a) Subject 82: female, alcohol user

(b) Subject 86: female, no alcohol usage

Figure A3: Functional connectivity matrix of two subjects with the highest and lowest model outcomes in CAP-C1.

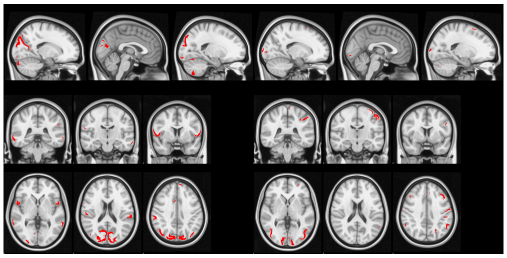

(a) C1-POS (b) C1-NEG

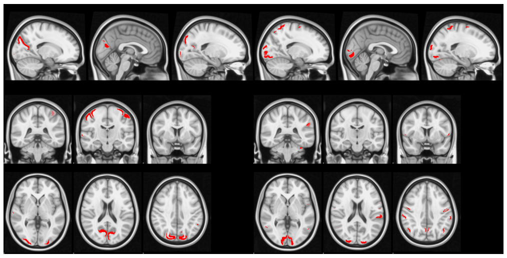

(c) C2-POS (d) C2-NEG

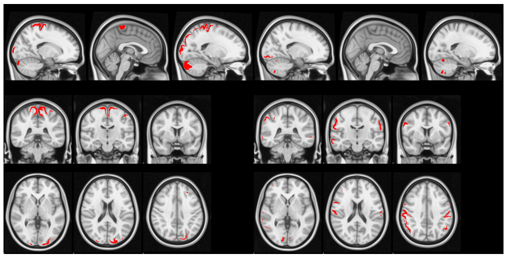

(e) C3-POS (f) C3-NEG

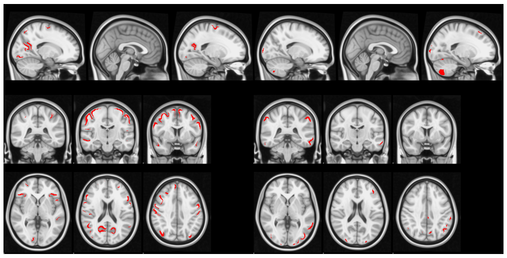

(g) C4-POS (h) C4-NEG

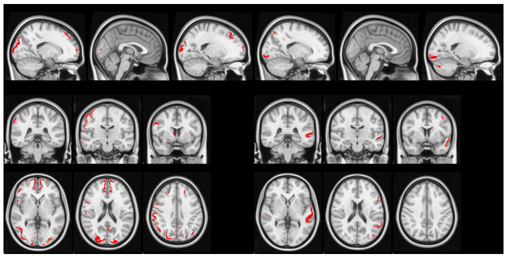

(i) C5-POS (j) C5-NEG

Figure A4: Reconstructed brain maps of the five components from the ICA-CAP approach. Regions with high positive (POS) and negative (NEG) loadings of each component are presented separately.

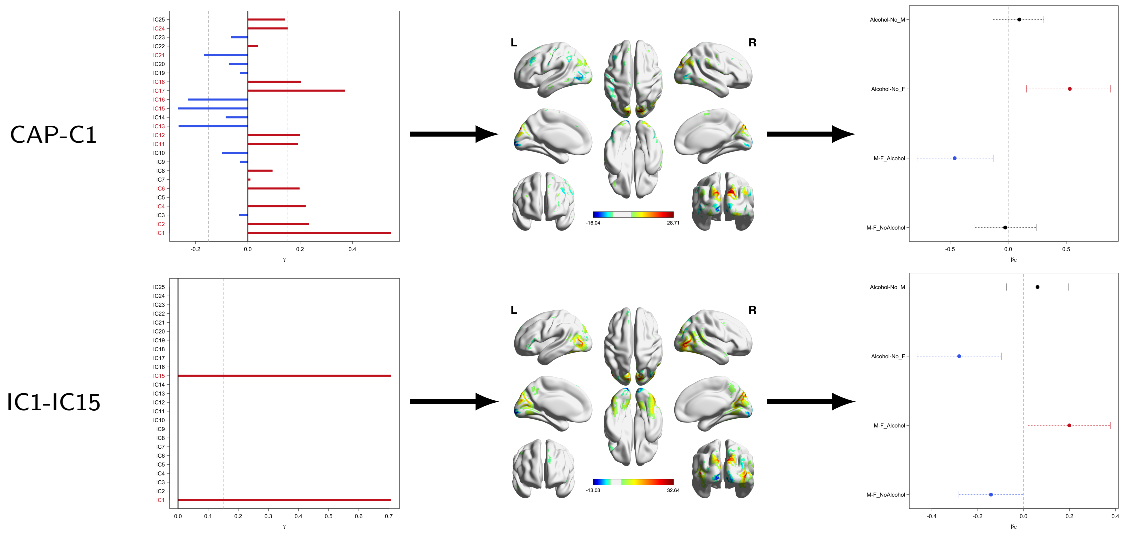

Figure A5: A comparison of CAP components C1 with element-wise regression of IC1 and IC15. Figures on the left panel shows the loading profile of the components/pairs, in the middle displays the corresponding brain map, and on the right presents the estimate of the four comparisons with 95% confidence intervals. For the element-wise approach, the brain maps are superposition of the two components with equal weights.

four components in REST2_RL. Figure A6 presents the chord diagram of the correlations between the components, where a connection indicates that the correlation between the two components is over 0.5. From the figure, the correlations between the first components identified across all sessions are relatively high (> 0.7), showing moderate reliability of the first component. Except for C5 of REST2_LR, the rest components are correlated with at least one component identified in another experimental session. This suggests a potential limitation of the proposed approach in reproducing components across sessions.

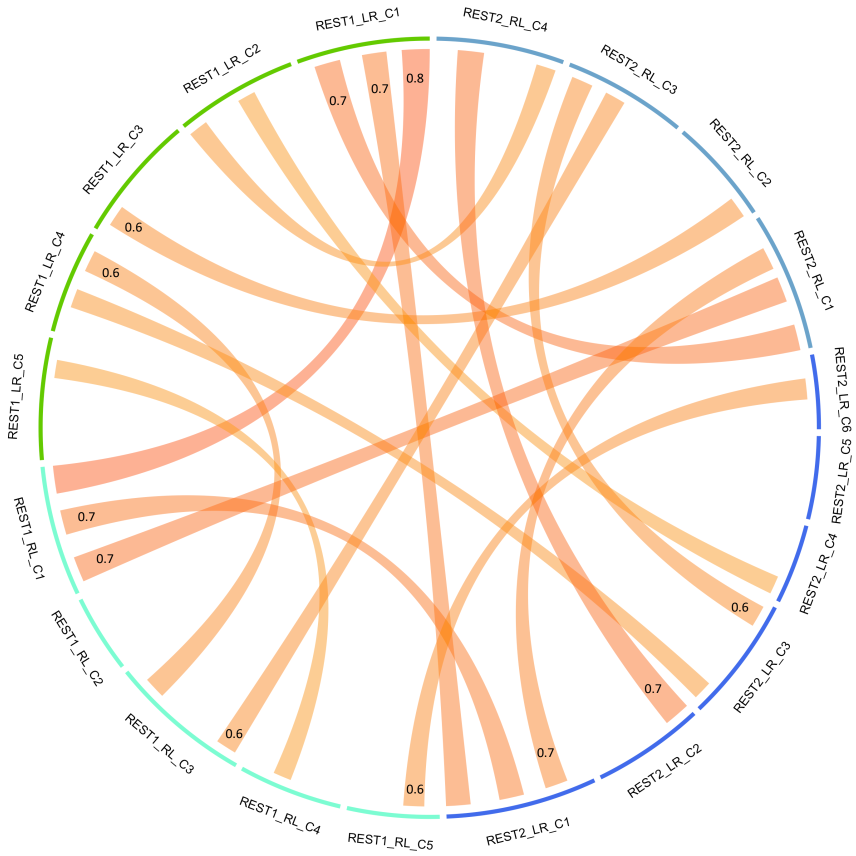

Figure A6: Chord diagram compares the similarity between the components identified from the four experimental sessions in the HCP data. A connection indicates that the two components have a correlation greater than 0.5. Correlations over 0.6 are labeled.
